# SIRIM Berhad: The Frontier in Medical and Health Technology

**DOI:** 10.21315/mjms2023.30.4.1

**Published:** 2023-08-24

**Authors:** Arshad Ahmad Sabirin

**Affiliations:** SIRIM Berhad, Selangor, Malaysia

**Keywords:** artificial intelligence, medical device, additive manufacturing technologies

## Abstract

With the world recovering from a public health disaster in the form of the COVID-19 pandemic and with political and social upheaval in the forms of wars such as in Ukraine and Sudan, localised fighting in various hotspots, the medical field faces huge challenges in addressing the needs of the various stakeholders. Still, these disasters represent opportunities to advance the new discoveries without compromising on the safety of the patients or general population. The COVID-19 vaccines were pushed through with great urgency driving on new discoveries of the genomic research, i.e. RNA based vaccines. This is complemented by the use of big data to monitor the disbursement of the vaccine to the general public. Unmistakably these new developments in tackling serious health disasters will lead to improvements on how the world tackle future crisis. Recent advances in artificial intelligence (AI), genomics discoveries and cell biology are driving research and fueling hope for the future. Amid this scenario of great upheavals and significant advances in technologies or emerging technologies for the health sector, SIRIM Berhad is increasing its preparedness in terms of development of new facilities and new competencies as well as building the platform for the communication of conventional health or medical technologies with other technologies particularly digital technologies to unlock the potential of emerging technologies in both medical and digital to develop novel solutions to future problems or challenges.

## Introduction

SIRIM Berhad is a premier industrial research and technology organisation in Malaysia, wholly-owned by the Minister of Finance Incorporated. With over 40 years of experience and expertise, SIRIM Berhad is mandated as the machinery for research and technology development, and the national champion of quality. SIRIM Berhad has always played a major role in the development of the country’s private sector. In the early decades after independence, Malaysia placed more emphasis on developing a quality culture as it seeks to establish itself as a trading nation by providing quality goods comparable to those from other developed nations (1). However moving forward it finds itself competing with other low-cost nations in providing the same quality goods and thus, realises the need to transform into a nation providing innovative products (1).

It is here that SIRIM Berhad has played a role and is still playing a role in the transformation of the country into an innovative nation. By tapping into our expertise and knowledge base, we focus on developing new technologies and improvements in the manufacturing, technology and services sectors. SIRIM Berhad Industrial Research is the frontier for technology innovations for various industries. SIRIM Berhad believes that through technological advancement, industries will see an increase in sustainability and in turn boosts the nation’s economy.

## Commercialisation of the Products

**Figure 1:**
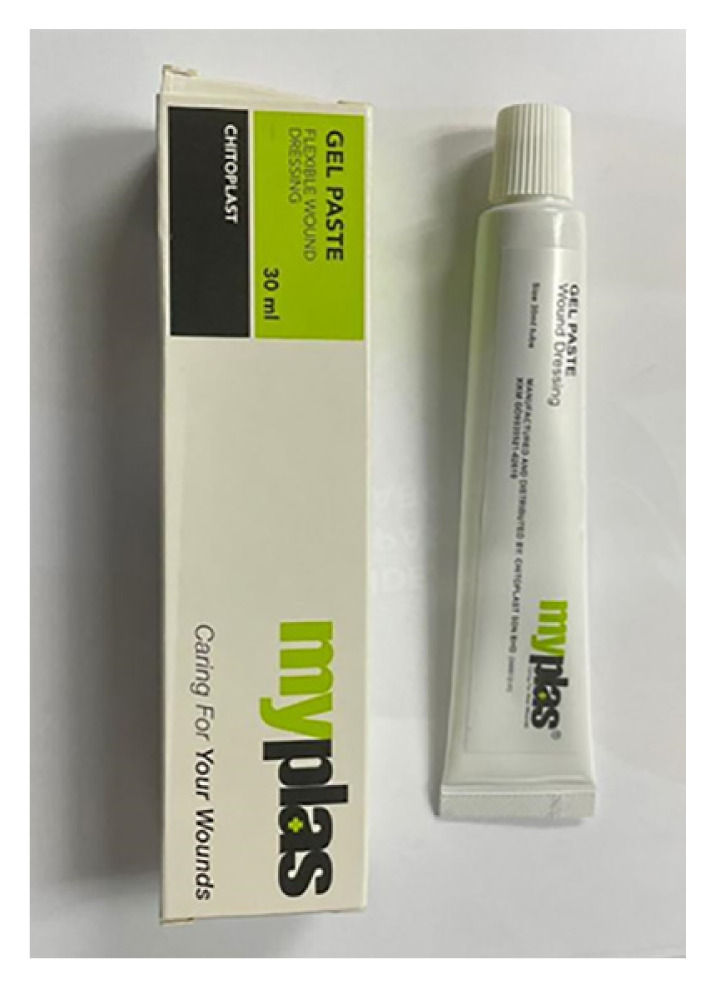
A range of wound management products

**Figure 2:**
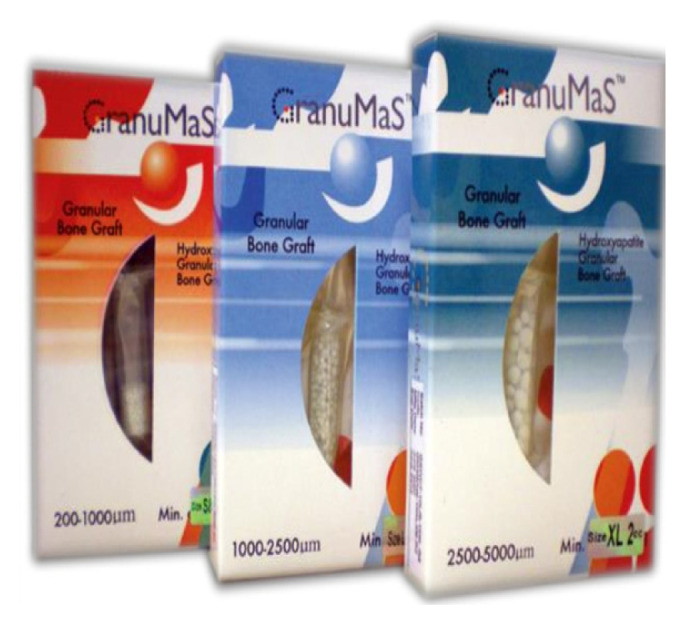
Hydroxyapatite based bone substitution product

**Figure 3:**
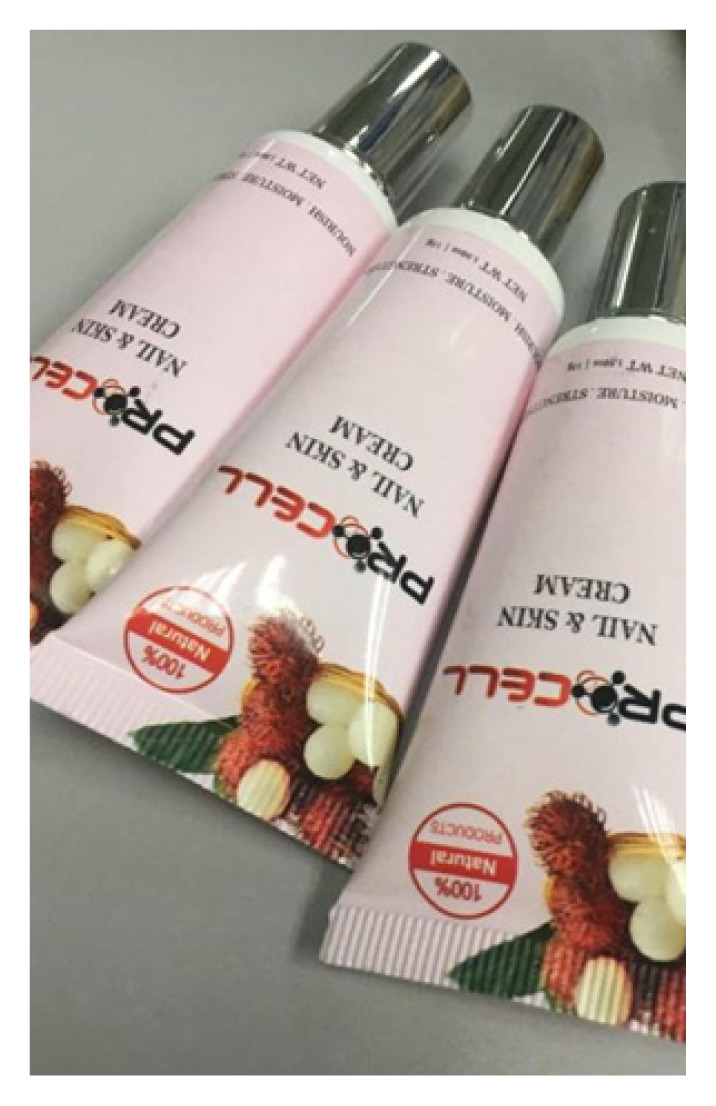
A natural based anti-fungal product

Among the industries where SIRIM Berhad is active is the medical and health industry where it is actively pursuing its interests in the areas of medical devices, traditional medicines, natural based drugs and pharmaceuticals. In this highly regulated industry, SIRIM Berhad has overcome the challenges to successfully place a few products in the market (2). Products in the market are wound management products (Figure 1), hydroxyapatite based bone substitution product (Figure 2) and natural anti-fungal product (Figure 3).

The experiences of going through the highly regulated health industry has enabled SIRIM Berhad to develop competencies to help the industry in developing an innovation culture. These competencies include advanced material technology, digitalisation, automation, the Industrial Revolution 4.0 (IR4.0), toxicology, manufacturing practices, regulatory testing and documentation and certification. SIRIM Berhad has identified and developed the ‘enabler’ facilities and expertise to steer our industry through novel product development and meeting the local and international regulatory framework requirements and establish Malaysia as a supplier of innovative medical and health products.

## Materials Testing for Medical Device

When it comes to the manufacture and fabrication of medical devices, high quality materials are necessary. The physical, chemical, thermal and mechanical properties of these materials can be used to characterise them. Materials testing is critical in the verification process. It determines the correct material and reliability for an application and satisfying regulatory and standard requirements as patient safety is crucial in medical device sector. SIRIM Berhad has aspirations to raise awareness about the need of materials characterisation and other testing in the medical device business.

All of the major players, such as the Association of Malaysian Medical Industries (AMMI), Malaysia Medical Device Association (MMDA), Malaysia Medical Device Manufacturers Association (PERANTIM) and other medical related associations would work closely with SIRIM Berhad in developing the industry further, citing that Malaysia has the potential to become a global player in the medical devices industry, particularly in the area of medical consumables.

At SIRIM Berhad in Kulim, Kedah, Malaysia we managed to get the ISO/IEC 17025: 2017 accreditation under the Advanced Materials Testing Laboratory for chemical, physical, thermal, biomechanical and microbiology fields of testing with SAMM no. 875. SIRIM Berhad Kulim has offered more than 10 ISO/IEC 17025 accredited tests since 2018. There are about 14 new testing methods which fall under the scopes of physical, chemical, mechanical, microbiology and thermal, and these tests are expected to cater to about 30% of the medical device companies located in the northern region (3).

In Shah Alam, SIRIM Berhad is able to add to the material characterisation testing by offering toxicology testing on the medical products, be it medical devices, pharmaceuticals, over-the-counter (OTC) products or traditional medicine. The tests performed follow the international standards or guidelines relating to each industry, ISO 10993 series for medical devices and the Organisation for Economic Coorperation and Development (OECD) Test Guidelines series for chemicals (intermediates) or pharmacopoeia for pharmaceuticals. To support the validation needs of medical research, SIRIM Berhad’s labs are accredited to the highest standards of OECD’s Good Lab Practice as well as ISO 17025 lab accreditation scheme to ensure that our results are fully validated and are internationally recognised (3).

## Contract Research Activities

In line with our philosophy of being a partner to industry in innovation, we also perform contract research for private industries both local and international. Due to the different expertise of medical research, partnership takes the form of each party performing specific activity in the research methodology. One such collaboration involves the evaluation of the in vivo efficacy of a pharmaceutical product while another collaboration studies the lab scale proof of concept of a cancer therapy for a US-based research company.

SIRIM Berhad’s lab was chosen as the testing lab to evaluate the process quality validation for one of the COVID-19 vaccines used in Malaysia making SIRIM Berhad a key contributor in the availability of the COVID-19 vaccines for the Malaysian public which helped the nation in controlling the spread of the disease. SIRIM Berhad is continually upgrades and enhances its facilities to offer our clients services which meets the ever evolving regulatory requirements (3).

## Current Research Project Activities

In terms of government research, SIRIM Berhad has carried out market-driven research and development (R & D) in biotechnology and biomedical to deliver outputs and products for future commercialisation. The details of the research projects are as follows:

### Digitalisation of Clear Aligners Production through Direct 3D Printing Technology

Direct 3D-printing clear aligner is a solution to make high value added clear dental aligners that provide an ideal platform for digital workflow, enabling more efficient and cost-effective fabrication of dental aligners. This new intervention will eliminate wastage by 50% and increase the efficiency by 50%. As the new technology reduces the waste in the manufacturing process, it could help solving tough problems of the humanity such as the consumption of the construction material resource, the energy consumption, and the environmental protection. Thus, it can reduce the price and more affordable to patients.

### Development of Metallic Implants Fabrication for Boosting Medical Device Industries in Malaysia

**Figure 4:**
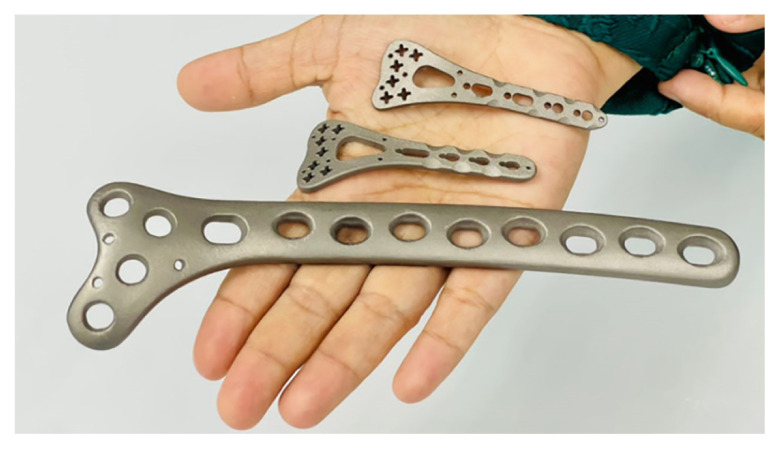
Metal 3D printing of titanium alloy for bone fracture

In an effort to address challenges from existing fabrication of implants, an innovative metal additive manufacturing using selective laser melting (SLM) technology for producing fracture fixation plates will be proposed as shown in Figure 4. This technology in pioneering rapid manufacturing process capable of producing fully dense metal parts direct from 3D CAD using high-powered laser. More generally, the unique capabilities of 3D metal printing enable new opportunities for customisation, improvements in product performance, multi-functionality and lower overall manufacturing costs.

### Transforming Orthopedic and Prosthetic Industry towards Digital Manufacturing using 3D Imaging and Additive Manufacturing Technologies

**Figure 5:**
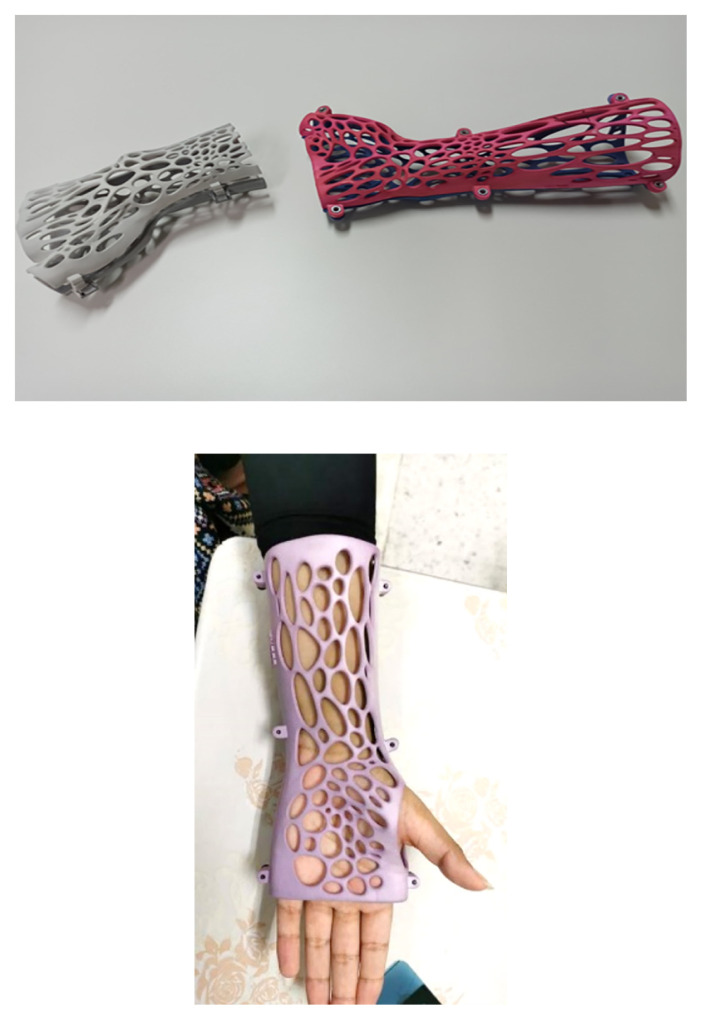
Examples of 3D printed prosthetic

**Figure 6:**
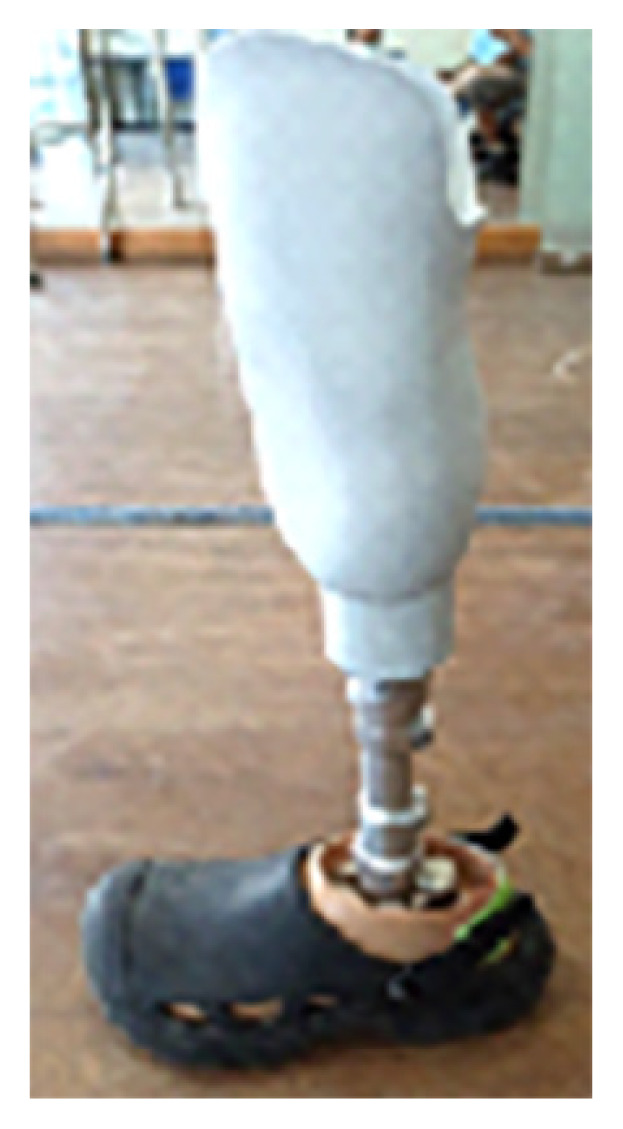
Example of orthopedic product

**Figure 7:**
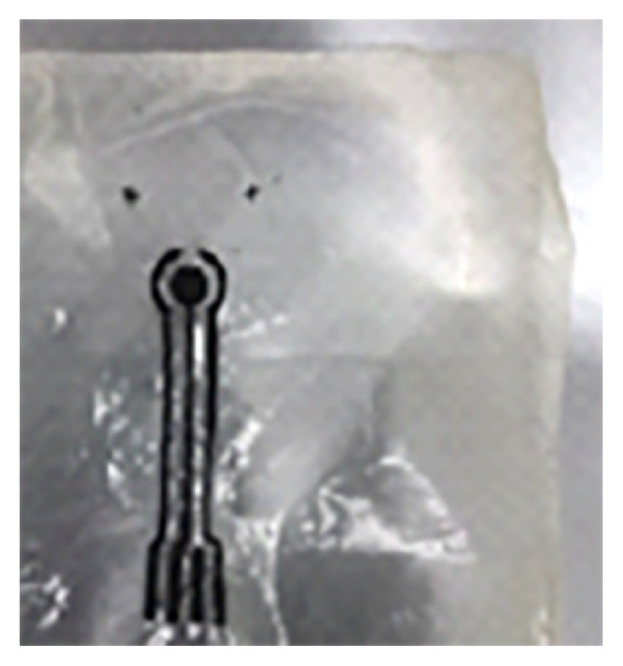
Prototype of wound plaster with the IoT device

Through the use of 3D digital imaging and additive manufacturing technology in the creation of orthopedic and prosthetic devices as shown in Figure 5, this project seeks to strengthen the medical device industry. With the aid of 3D scanning, 3D computer-aided design (CAD) and 3D printing (additive manufacturing) technologies, SIRIM Berhad is currently collaborating with a few local companies to develop below-knee prostheses (sockets) and the prototype of wound plaster with the Internet of Things (IoT) device (Figures 6 and 7). The project will leverage on existing 3D printing capabilities in SIRIM Berhad, with the expansion and acquisition of equipment and facilities to offer easy access to small and medium enterprises (SMEs).

### Microbiomes and Post-biotic Bioactive: New Dimension in Beauty Care and Healthcare

**Figure 8:**
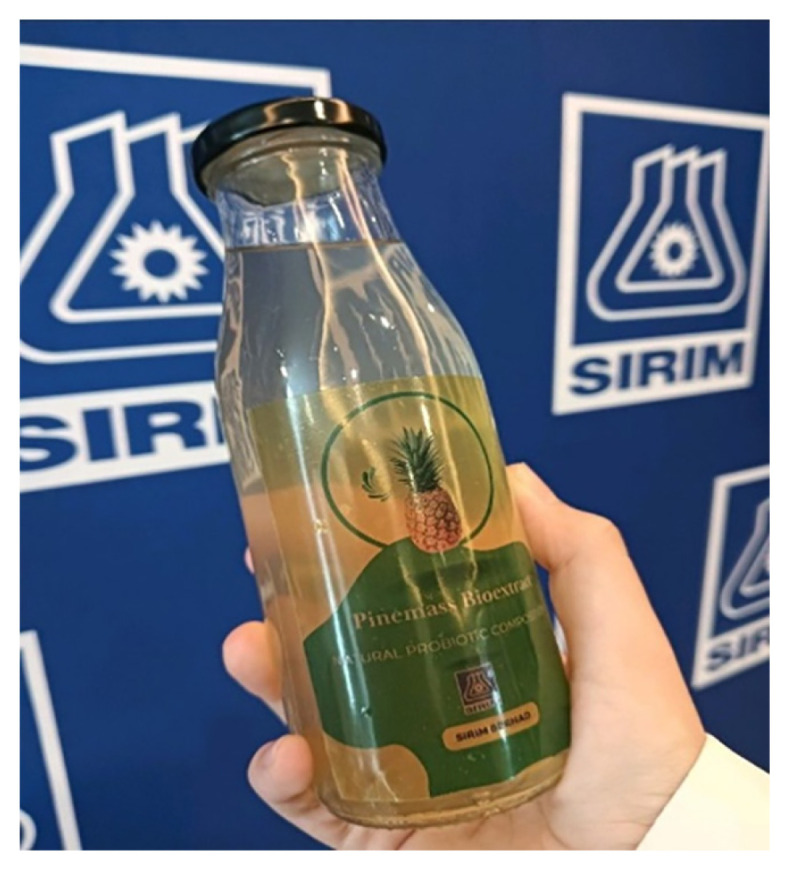
Pinemass, a clarified post-biotic extract produced through fermentation process of pineapple biomass

The holistic approach to beauty and health has gained significant traction, with a growing belief that achieving beautiful skin requires a healthier lifestyle encompassing an optimal diet, supplements and physical activity. Within this context, the emergence of microbiome skincare has created a new category that combines elements of cosmeceuticals and nutraceuticals to promote beauty from within and maintain a healthy skin microbiome. These products have the potential to improve skin health and prevent issues like dryness, aging and acne. Microbiome skincare often incorporates a combination of probiotics and prebiotics, which can generate post-biotics—bioactive compounds that promote skin health—in both topical and oral products. We have achieved successful commercialisation of our proprietary post-biotic fermented extract derived from pineapple biomass, which we refer to as Pinemass Bioactive, to industrial taker as shown in Figure 8. This extract is specifically developed for cosmeceutical applications, offering a range of beneficial effects for skincare and beauty purposes. Furthermore, we have ensured that the product adheres to regulatory standards and meets the requirements for halal and safety, making it suitable for consumers who prioritise these considerations.

The nutraceutical and pharmaceutical applications of our advanced microbiomes and post-biotic extract is also promising. Our ongoing research has yielded intriguing results, suggesting that the post-biotic extract exhibits beneficial properties such as enhancing the immune system, and demonstrating potential as an anti-asthmatic and anti-viral agent. Our current endeavours in the realm of microbiome skincare represent just the beginning of our journey towards developing even more advanced active ingredients for the promotion of health and beauty. We are committed to upholding environmental sustainability by integrating environmental, social and governance (ESG) principles into our inventions and product development processes. Our aim is to create innovative solutions that not only deliver exceptional results but also contribute positively to the environment and society.

## Establishment of Medical Device Innovation Centre

Malaysia is evolving as a hub of medical devices manufacturing in the Asia pacific region with more than 200 medical devices manufacturing companies. The industry is capital- and technology-intensive and employs over 70,000 people. Across the manufacturing value chain, the medical device industry has moderate capabilities in component manufacturing, manufacturing and assembly and post-sales services which are needed to be strengthened to advance the industry.

**Figure 9:**
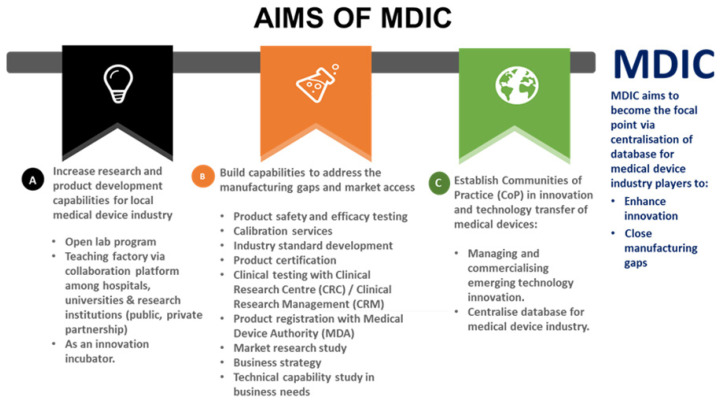
The aims of establishment of MDIC

The level of R&D spending on medical devices companies is low (4). Most Malaysian companies spend about 1% of revenue on R & D expenditure as compared to 3%–9% by companies in peer countries. In this scenario coupled with our experiences of working in the medical devices industry in various capacities, SIRIM Berhad has established the Medical Device Innovation Centre (MDIC) to enhance its contribution to the industry in Malaysia by addressing manufacturing gaps through automation, accredited medical device testing services to meet regulatory requirements, product certification, research, development, innovation and commercialisation (3) as shown in Figure 9.

MDIC platform is intended to advance the medical device industry in Malaysia through technology innovation, R & D, commercialisation, talent and capacity building. The main focus of the MDIC is to address the key challenges in medical device industry, particularly the innovation ecosystem, through data gathering and engagement with major players and stakeholders through a series of workshops and forums and provide the way forward to close the gap between industry and SIRIM Berhad.

## Moving Forward

**Figure 10:**
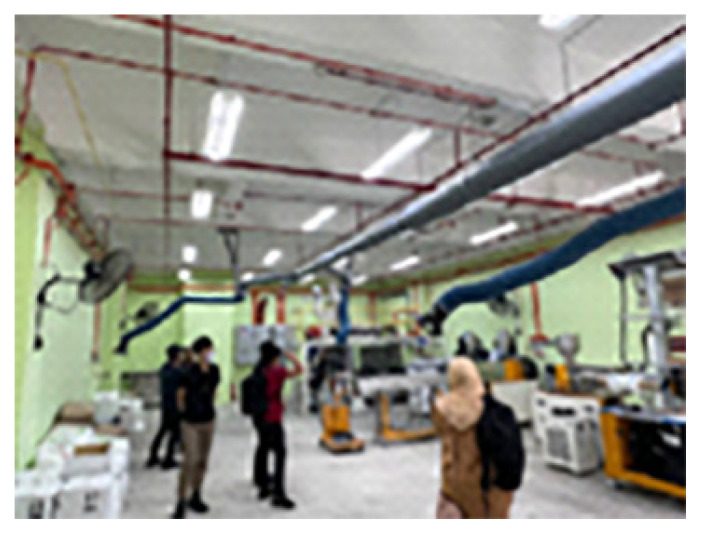
Pilot plant facilities at SIRIM Berhad

As stated above, artificial intelligence (AI), genomics discoveries and cell biology are some of the frontier technology research being performed in the health sector. It is imperative that Malaysia is active in these research areas to ensure our people benefits from frontier technologies in the shortest possible time. As a research institution SIRIM Berhad that has the capability of develop products using a pilot plant scale as shown in Figure 10 is also actively involved in partaking in activities related to these fields. For example the toxicology lab is pursuing the ‘new approach methodologies’ to toxicology testing which exploits the recent advances in cell biology to migrate from animal to non-animal testing. The ‘new approach methodologies’ also incorporates technologies like the organ-on-a-chip which can be used to simulate the effects of materials/samples/drugs on human organs without actually using human patients. It is believed that this methodology is more accurate than using the animal models especially the small animal models for gauging the effect of substances on humans/organs.

**Figure 11:**
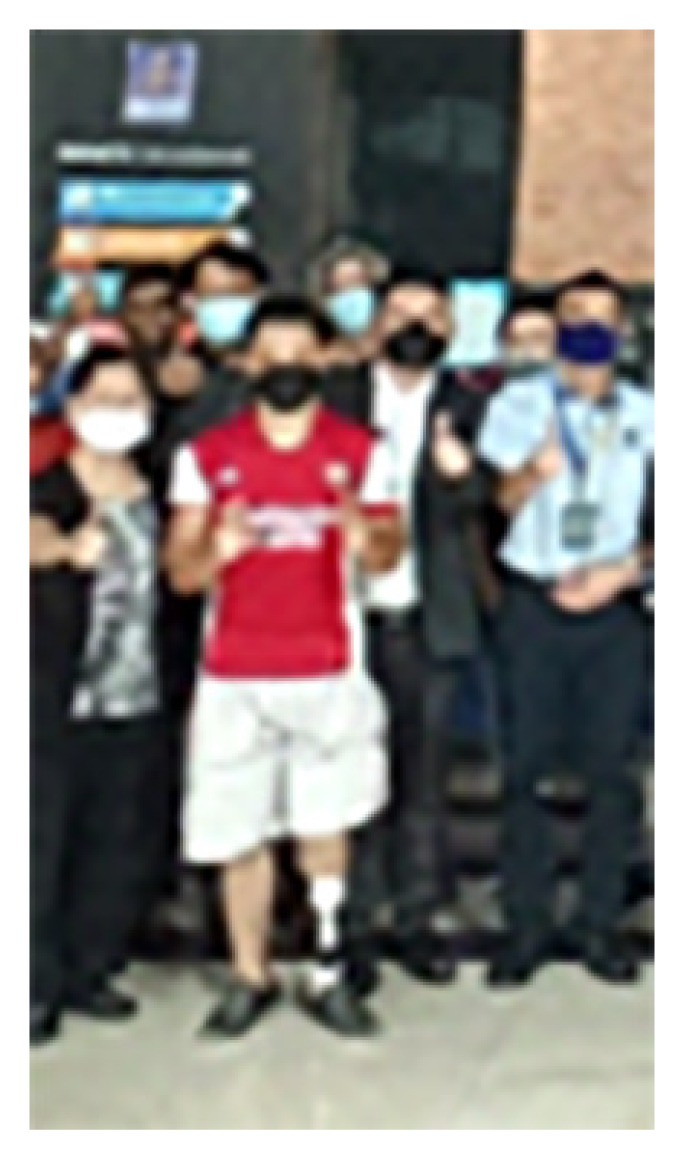
SIRIM Berhad products demonstrated a significant impact to the community

Another area which is of interest to SIRIM Berhad is in the application of AI to health research. AI is a subject close to SIRIM Berhad’s heart but its application has been limited to manufacturing technologies. However, the power of AI goes way beyond any specific industry and it is in SIRIM Berhad’s strategic plan to invest on developing the application of AI in the health industry. Of particular interest is in the application of AI for predictive outcomes of health intervention or in research methodologies. The genesis of the application of AI could be in the automation and digitalisation of some of the processes in the health industry especially where a lot of data needs to be collected and analysed to be followed by the application of robotics technology which culminates in a greater diffusion of more complex AI technology for a greater leverage of frontier technology in the medical and health industry (5) (Figures 10 and 11).

## Conclusion

SIRIM Berhad aspires to be a partner to all the stakeholders in Malaysia’s health industry in ensuring a delivery system of the highest quality health service to the Malaysian community. SIRIM Berhad must adapt and provide scientific and technological leadership in a changing national and global environment. It further engagements with the stakeholders in the Malaysian health industry, and we are confident this aspiration is achievable in the near future.
